# Self-care with non-prescription medicines to improve health care access and quality of life in low- and middle-income countries: systematic review and methodological approach

**DOI:** 10.3389/fpubh.2023.1220984

**Published:** 2023-09-13

**Authors:** Uwe May, Cosima Bauer, Anissa Schneider-Ziebe, Chiara Giulini-Limbach

**Affiliations:** ^1^May & Bauer GmbH & Co. KG, Bad Honnef, Germany; ^2^Faculty of Economics and Management, Hochschule Fresenius University of Applied Sciences, Wiesbaden, Germany

**Keywords:** self-care, low- and middle-income countries, non-prescription medicines, access to health care, economic methodology

## Abstract

**Objectives:**

This study aims to develop a structured framework to capture beneficial effects and determine the value of self-care for individuals and society in low- and middle-income countries (LMICs). A special focus is placed on self-medication with non-prescription medicines.

**Methods:**

PubMed, Google Scholar and websites of associations or organizations were systematically searched for economic studies on self-care and self-medication published between 2000 and 2021. The insights gained from the literature review were incorporated into the development of a decision tree model.

**Results:**

The literature review revealed a lack of research and available data on the role and value of self-care in LMICs. To help close the research gap a methodological framework was developed that defines different settings of self-care, their effects and relevant outcomes and allows a quantification with regard to self-medication in LMICs.

**Conclusion:**

Self-care offers individuals a convenient and reliable way to take care of their own health, especially in LMICs where access to health services can be challenging. In particular it is crucial to improve individuals access to clinically effective, safe and reliable non-prescription medicines.

## Introduction

The World Health Organization (WHO) reports that at least half of the world's population lacks access to essential healthcare (1). This barrier to healthcare is driven by shortages in healthcare providers, a lack of healthcare facilities and high costs of healthcare services (2). Therefore, according to the WHO, self-care interventions belong to the most promising approaches to improve health and wellbeing, both from a health systems perspective and for people using these interventions. Self-care as a broad concept encompasses hygiene, nutrition, lifestyle, environmental factors, socioeconomic factors and self-medication (3).

The self-responsible use of non-prescription medicines [also referred to as over-the-counter (OTC) medicines], so-called self-medication, globally plays an important role as one kind of intervention within the broad concept of self-care. Self-medication may often be supplemental to services offered by healthcare systems or, more likely, may even be the only available option for individuals to gain access to healthcare in low- and middle-income countries (LMICs). It enables individuals to manage self-treatable conditions without the need to visit a physician or an emergency department (ED) (4). However, studies and data on the socio-economic value of self-medication in LMICs are few so far. Existing studies focus mainly on high-income countries. The methods used there are not suitable for capturing the value of self-care and self-medication in LMICs. This gap is to be closed with the conceptual considerations presented here.

The availability of effective OTC medicines and information on their use is an enabler for self-medication. OTC medicines are legally restricted by national or supranational regulatory authorities to products that can be appropriately self-selected and used by consumers based on information provided by e.g., product leaflets or pharmacists (5–9). Against this background, OTC medicines can be considered as essential tools in the context of self-treatable health conditions (STCs). The supervision of a healthcare practitioner is typically not required. The importance of this treatment option and low-threshold access to care in general has been demonstrated by previous research, which suggests that treatment of STCs, when necessary and appropriate, should be the primary treatment strategy over foregoing treatment (10–12).

This study aims to develop a structured framework to capture beneficial effects and determine the value of self-care for individuals and society in LMICs. The classifications according to the World Bank serves as an orientation for the delimitation of LMIC (13). A special focus is placed on self-medication with non-prescription medicines. A clear definition of self-care concepts including self-medication and outcome measures may enable to highlight the benefits that can be gained by individuals in LMICs if they self-medicate. Based on this framework, the approach offers the opportunity to quantify self-care effects by focusing on self-medication with OTC medicines. Thus, the framework is intended to support those conducting self-care analyses as well as stakeholders like WHO in interpreting and applying the evidence as a basis for health political decisions and measures for self-care encouragement.

## Methods

A two-step methodological approach was applied to develop a framework to determine the social and economic value of self-care in LMICs. First, economic analyses on self-care in general and specifically on self-medication were systematically reviewed to gain an overview about the methodological approaches applied and outcomes considered in economic studies on the value of self-care. Next, a decision tree model was constructed to define self-care concepts which practically represent different behavioral models and to identify the relevant costs (resource use) and consequences (outcomes and effects) associated with these behavioral models.

### Systematic literature review

A systematic literature review was conducted following the Preferred Reporting Items for Systematic Reviews and Meta-Analyses (PRISMA) guideline. PubMed database and Google Scholar search engine were searched for peer-reviewed studies. Gray literature, including reports and white papers, policy documents, fact sheets, newspapers and other reports from consumer health or self-care associations or organizations were identified through targeted website searching, citation searching and gray literature search engines (e.g., Think Tank Search, Gray Literature Report, Trip Medical Database). A strong focus was placed on gray literature sources as guidelines for conducting systematic reviews of evidence from economic evaluations recommend the inclusion of gray literature to reduce bias, avoid the omission of potentially relevant work and improve the quality of the review synthesis.

Keywords relating to prescription (Rx) and OTC medicines, including “Rx,” “OTC” and “non-prescription” were included as search terms in addition to the adjectives, “change,” “switch” and “reclassification” to identify any Rx-to-OTC switches. The terms “self-care,” “self-treatment,” “self-medication,” “OTC” and “common health condition” restricted the search to evidence on self-care that is specifically related to the treatment of STCs. The search also included the terms “economic,” “social,” “cost,” “impact,” “benefit,” “analysis” and “potential” to identify comparative research on the topic of self-care.

The search was limited to articles published between 2000 and 2021. The criteria for inclusion (determination of economic or social value of self-care focusing on OTC medicines or STCs from countries or regions around the world, English and non-English, abstract and full-text studies, peer-reviewed and gray literature) and exclusion (studies focusing on self-care of chronic disease, study protocols and case reviews, non-health economic studies and studies that described self-care in the context of chronic disease) of studies was established a priori.

Studies were screened by two independent reviewers and findings from eligible studies were extracted: geographic scope, aims/objectives, type of economic study, methodology, scenarios considered, stakeholder perspective, costs included, model assumptions (where applicable), social and economic findings. The studies were categorized using an inductive approach of qualitative synthesis to derive common themes (14). A quantitative data synthesis was not possible due to the heterogeneity of the study designs, the interventions and the outcomes measure among the included studies (15). Therefore, a narrative summary was carried out.

The quality of the studies was assessed using the Joanna Briggs Institute (JBI) Critical Appraisal Checklist for Qualitative Research (16). Ten criteria were assessed by answering “Yes,” “No,” “Unsure,” and “Not Applicable” to questions pertaining to congruity between the research methodology and research questions, as well as methods used to collect data. A score of 1 (minimum) to 10 (maximum) was assigned to each study based on the number of “Yes” answers recorded.

### Decision analytic modeling

According to the two-step methodological approach, a decision tree (i.e., a simple structural decision analytical model) was developed to simulate patient behavior and the consequential results of their decision-making in the cases of an acute or no acute STC under real-life conditions (17).

The decision tree was fed with data that was extrapolated from the systematic review on the economics of self-care and self-medication with OTC medicines (18, 19).

**Figure 1:**
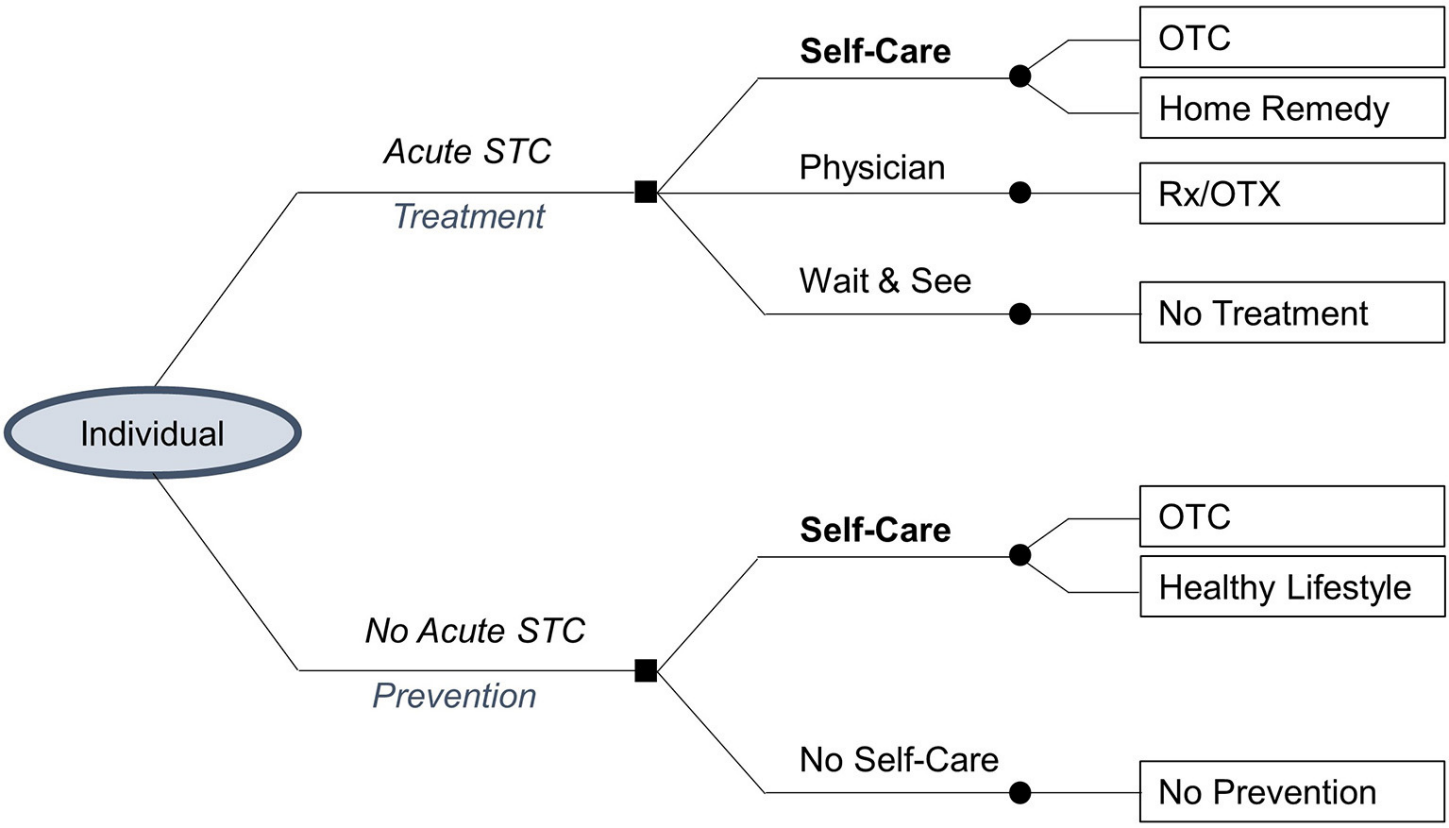
Decision tree.

In Figure 1 the square nodes at the start of the decision tree represent the decision between alternative treatment pathways for a case of acute or no acute STC. The circular nodes indicate the possible alternative treatments that a patient may choose (self-care, physician visit, wait and see, no self-care). Despite the options to practice self-care or visit a physician to actively treat (or prevent) an STC, a patient may still choose to (temporarily) do nothing. Each decision leads to possible treatment tools (OTC medicines, home remedy, prescription-only medication, no treatment, healthy lifestyle, no prevention). The pathways (“branches”) following each node represent mutually exclusive events that each lead to distinct outcomes for the patient.

The decision tree is qualitatively analyzed to determine the effects that may occur when a patient chooses the intervention treatment pathway or self-care over a comparator treatment pathway (20). In this analysis, the intervention in the treatment pathway is self-care by using OTC medicines, as other effects, e.g., due to hygiene measures, are hardly reliably quantifiable. The comparator treatment pathways in the case of an acute STC (physician and wait and see) were considered as the only choices when appropriate OTC medicines are not available. In the case of no acute STC, which is the preventive pathway, no self-care is the comparator pathway. Opportunity costs are determined according to this approach under the premise that the use of OTC medicines to treat an STC will lead to the same outcomes as a physician visit (21). This qualitative analysis was complemented by evidence from the systematic literature review.

## Results

### Summary of systematic literature review findings

**Figure 2:**
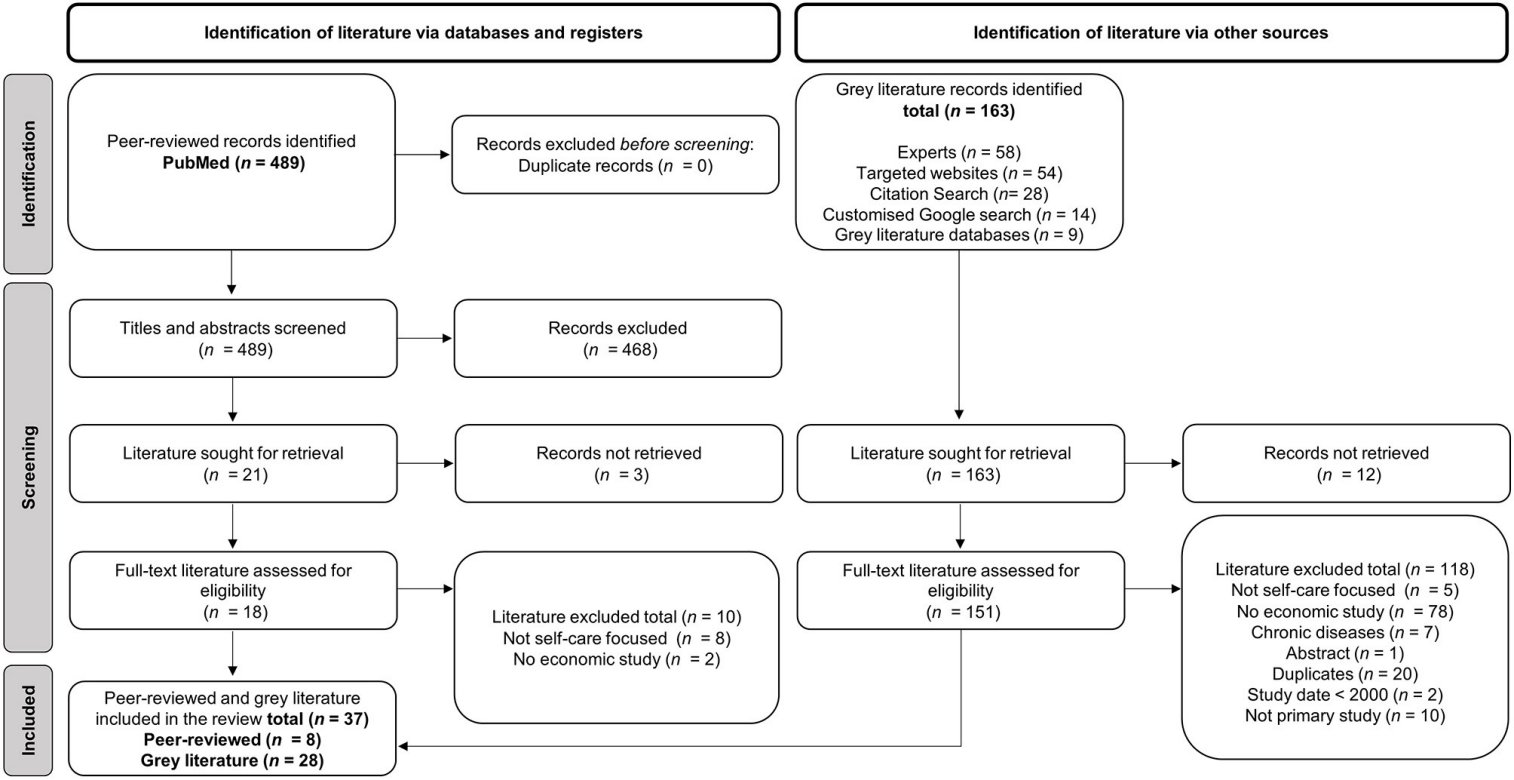
PRISMA-P flow diagram.

The 489 peer-reviewed studies and 163 results for gray literature were initially identified. the titles and abstracts of these studies were screened and a total of 169 studies were reviewed in full. The 128 of these were excluded for the following reasons: did not focus on self-care, focus on self-care of chronic diseases, not a primary study, duplicate, or no economic evaluation conducted (see Figure 2). Thirty-six studies were selected for inclusion and received a critical appraisal score between 77.8 and 100.0%

The majority of included studies focuses on Europe (19) and North America (13). Country-specific studies on self-medication were identified for Austria (1), Germany (4), Italy (1), Spain (1), Switzerland (1), the UK (8), India (1), Australia (2), Brazil (1), and Mexico (1) (21–38). Two multi-country studies were found, including a study conducted in 2004 on seven European countries and a study published on five Latin American countries in 2019 (39, 40).

Almost 70% of the studies selected for inclusion examined OTC medicines as intervention in comparison to prescription-only medicines or alternative treatment-seeking behavior including physician visits, walk-in clinics and ED visits.

Although available research demonstrates that self-medication with OTC medicines is a cost-effective solution to improve access to healthcare in both high-income countries (HIC) and LMICs, the literature review revealed that the overall number of studies on the value of self-care in LMICs is very limited. Focusing on economic studies on self-care in LMICs, a study on self-care in the context of chronic health conditions in Vietnam was published in 2020 and in the following year, evidence on the economic potential of self-medication for common ailments in India was published (22, 41). Nevertheless, the majority of studies describing the economics of self-care and more specifically of self-medication focus on high-income countries and aim to highlight how self-care practices with OTC medicines can alleviate healthcare system overuse. For example, a European-wide study found that self-medication can reduce the number of physician visits while generating savings of ~23.3 billion euros per year in medical services and products (42). This substitutive effect that occurs when individuals practice self-care or self-medication instead of visiting a physician for an STC is somewhat irrelevant in LMICs. In a scenario where a physician visit is no available option or access to medical care is severely restricted, the study approach used usually in HICs is obviously not appropriate. This means that the established study approaches cannot depict a situation in which the only available alternative to self-treatment is foregoing treatment altogether. For this reason, these approaches are not transferable to the prevailing conditions in LMICs. The issues mentioned result in healthcare system underuse and in self-care often being the only means to increase the active treatment of existing STCs and to encourage preventive behavior in LMICs (43). To be able to also draw conclusions about the effects of self-medication for these countries, study approaches that are based on an extended decision-analytical approach are required.

### Decision tree model description and self-care concepts

**Figure 3:**
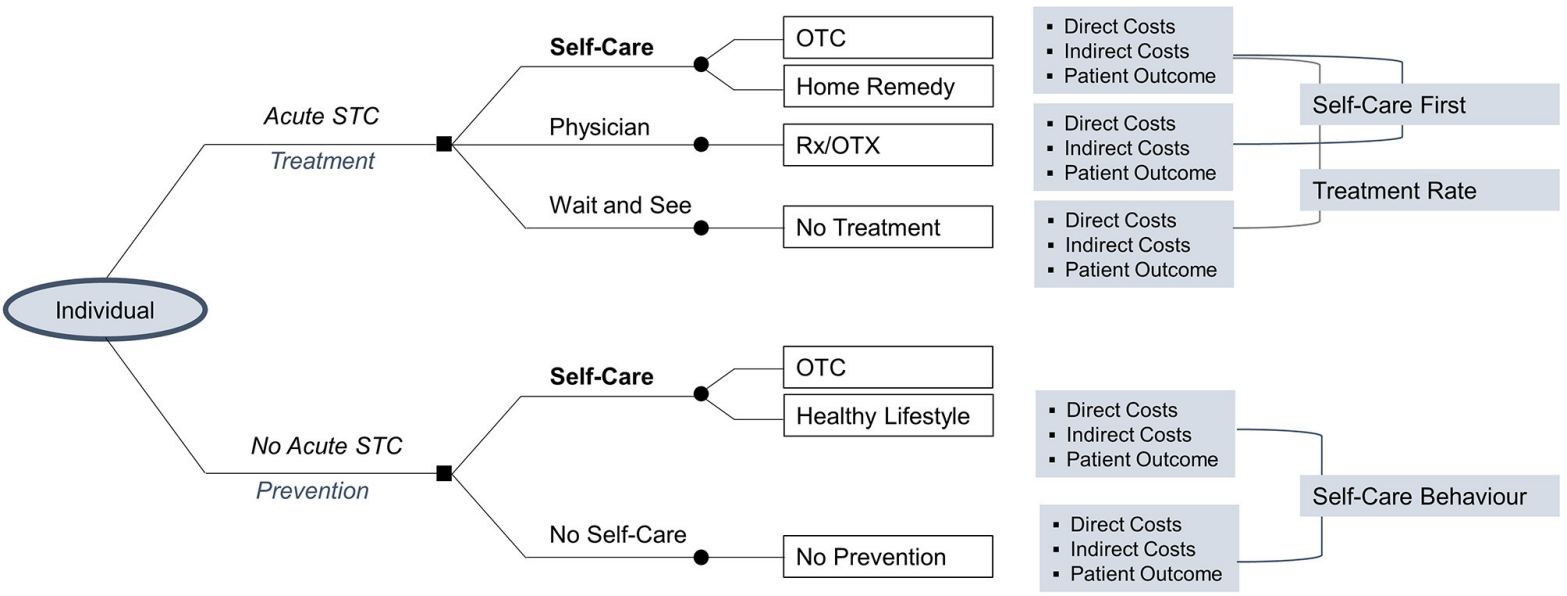
Decision tree analysis.

An analysis of self-care against the two alternative treatment options of a physician visit as well as wait and see for an acute or no acute STC reveals three main concepts in the context of self-care. These are described as “Self-Care First,” “Treatment Rate” and “Self-Care Behavior” (see Figure 3).

In the case of an acute STC, an individual has three possible choices including self-care, a physician visit or wait and see (i.e., do nothing). Each choice leads to corresponding cost and benefit outcomes. A substitutive effect is observed when an individual chooses to use OTC medicines or home remedies to manage an acute STC (i.e., self-care) over a physician visit. This concept is here referred to as Self-Care First. Alternatively, when an individual chooses to use OTC medicines or home remedies for an STC rather than doing nothing, this may increase the number of individuals that receive treatment for an STC. This behavioral concept is here designated as Treatment Rate.

In the case of no acute STC, the effect observed when an individual chooses to actively engage in preventive forms of self-care activities rather than doing nothing can be characterized as an increase in preventive health behavior and is therefore called Self-Care Behavior.

Each of the three self-care concepts can be characterized by a set of tools, their main target and types of social values (Table 1).

**Table 1:** Three main self-care concepts.

**Self-care concepts**	**Definition**	**Tools**	**Main target**	**Social value**
Self-care first	Choosing self-care with OTCs instead of a physician visit	OTCs (medicinal products)	Symptomatic relief	• Same outcome • Freed-up resources (e.g., physician visits)
Treatment rate	Choosing self-care with OTCs instead of deciding to “wait and see”	OTCs (medicinal products)	Symptomatic relief Prevention	• Improved outcome • Decreased/avoided disease burden and cost
Self-care behavior	Choosing pro-active self-care behavior	Healthy lifestyle, home remedies, OTCs (for preventive use), hygiene	Prevention	• Improved outcome • Quality of life • Improved public health

The social values associated with the *Self-Care First, Treatment Rate*, and *Self-Care Behavior Concepts* can be divided into the three basic categories *quality of life (A), welfare (B)*, and *cost containment (C)* that are of greatest significance for each concept and are described below.

Within the *Self-Care First Concept*, the choice of self-care as the first treatment option predominantly involves the use of OTC medicines. As these products are intended to help patients resolve symptoms of self-diagnosable and self-limiting minor ailments without healthcare practitioner supervision, it is expected that both self-care and physician treatment choices will result in equivalent patient health outcomes. In individual cases it may happen that a patient receives incremental health-related benefit by choosing one or other of the two alternative treatment paths. However, the most significant benefits from choosing self-care instead of a physician visit are resource savings for national healthcare systems. Moreover, considering the opportunity costs of a physician visit when an individual seeks treatment for an STC, time savings for patients and physicians are realizable. The latter is therefore particularly meaningful, as physician capacities are usually scarce and can then be used to benefit those patients who have a serious illness that requires more complex treatment.

Within the *Treatment Rate Concept*, the choice of self-medication with OTC medicines, instead of doing nothing, implies that the individual actively treats an existing STC. Individuals can improve their quality of life by using OTC medicines as they provide symptomatic treatment and shorten the duration of the STC. Since OTC medicines often allow for fast symptom relief and the return to or continuation of paid employment, the amount of lost wages can be minimized or avoided altogether. The ability to work may be a determining factor in meeting basic living necessities (e.g., food and housing) in disadvantaged populations. Thus, quality of life and welfare considerably impact the social value of care in the Treatment Rate Concept.

Within the *Self-Care Behavior Concept*, quality of life, welfare and cost containment all exhibit a substantial effect when an individual undertakes preventive behavior. However, the quantification of outcomes related to Self-Care Behavior is beyond the scope of this study and the approach to determining the value of self-care within this concept is not analyzed further in this study. The reason for this is insufficient knowledge regarding the extent to which health promotion, disease prevention and control as well as other forms of self-awareness as individual prevention actually occur and how to distinguish these behaviors from normal lifestyle behaviors in terms of definitions and scope.

### Method components and data needs

Based on the self-care concepts and three basic categories as defined in this study (described above), six specific self-care effects were identified. The quantitative values of these effects can first be determined on an individual level and added up to obtain the value of self-care on a societal level. In addition to the perspective of the patients, the national healthcare system and national economy perspectives were found to be relevant when determining the value of self-care. In the following, the major self-care effects are described according to resource inputs associated with an individual's treatment decision. The stakeholder perspectives that should be adopted when quantifying and interpreting the value of the self-care effect are also specified.

#### Quality of life

Quality of life encompasses one aspect of the value of self-care associated with improving an individual's health, which is facilitated by broader, more convenient and/or faster access to healthcare through self-care practices (10). Symptoms associated with self-treatable conditions may negatively impact overall quality of life, especially if they are poorly treated or untreated. Impact on quality of life includes sleep, work performance, daily activities and social relationships. By actively managing symptoms of an STC, individuals can regain time for social activities, leisure time, as well as the ability of undertake normal daily activities and household, school or paid work (44, 45). Quality of life is measured on a scale of 0 (death) to 1 (full health) as applied in the established approach of quality adjusted life years (QALYs) (46).

#### Welfare

##### Individual productivity

Adopting the consumer perspective, the time needed for a physician visit can be avoided if an existing STC is treated with OTC medicines. Moreover, convenient access to OTC medicines may lead to sufficient and fast reductions in symptoms and enable those with STCs to minimize or even eliminate the time spent away from work, school or other daily activities (e.g., household or child care). To calculate productivity, data is needed on the health status of an individual and the number of days they are absent from work due to a case of STC that is untreated respectively treated with OTC medicines. This could include the number of sick leave days taken by an individual with an STC provided through administrative sources (e.g., insurance agencies) or self-reported absence from work through questionnaires or surveys. The amount of time needed to purchase OTC medicines from e.g., the pharmacy or drugstore should also be considered in the productivity calculations.

##### Social welfare

Welfare refers to the state of the overall societal economic situation. Self-care enables individuals to recover faster from their STCs and resume their daily tasks, including personal responsibilities and employment. Therefore, a reduction in the number of sick days leads not only to increased productivity, but also to greater economic welfare in terms of avoided wage losses. In this context, the positive effect on an individual level contributes to the overall societal welfare. Data required to determine the magnitude of this effect includes real income data, whereby a positive change in per capita income or GDP indicates an increase in welfare.

#### Cost containment

##### Monetary savings for national health care systems

This effect captures the monetary savings that can be achieved through self-care practices from the perspective of a national healthcare system or national economy when an individual practices self-medication. These savings arise from more efficient use of health resources when unnecessary physician or ED visits are avoided and when available OTC medicines offer effective treatment in lieu of prescription medicines that may be reimbursable. Hence, the direct cost components of a physician visit and the use of OTC medicines in the context of self-care must be considered. At the national level, the average price of prescription medications and the cost of a physician visit could be based on the reimbursement amount by payers (e.g., public health services under government administration or health insurance companies).

##### Patient time savings

The perspective of patients when they choose physician treatment is adopted for this effect. There are four key considerations related to patient time: (1) waiting time to get a physician appointment, (2) travel time to reach a physician, (3) waiting time at the physician's office, (4) duration of the consultation.

##### Physician time savings

This effect is concerned with the amount of time that a physician requires to attend to a patient presenting with an STC. According to the definition of an STC, this is a health condition that is self-diagnosable and can be treated with OTC medicines without the supervision of a healthcare practitioner. Thus, if an individual chooses to practice self-care for a case of STC, time is freed up for the physician to attend to patients with more severe and urgent health conditions or they are able to gain leisure time. The time-related considerations include the duration of a consultation, the buffer time in between patients and the proportionate time per case needed for administrative tasks.

## Discussion

The systematic literature review findings highlight gaps in economic evidence on self-care and self-medication in LMICs. Despite limited evidence for LMICs, the available evidence demonstrates that self-medication can be of significant value to each relevant stakeholder in healthcare systems worldwide. Additionally, no consistent methodologies for determining the value of self-care in LMICs can be identified in the systematic literature review, which contributes to the fact that many studies focus predominantly on monetary savings while overlooking other significant effects of self-care such as welfare and quality of life. Based on these findings, the present study set out to close the research gap by developing a new framework to determine the value of self-care with a focus on self-medication on a global scale and to highlight the benefits of self-care for LMICs in particular.

The decision analytic approach enables the integration of evidence from multiple sources and of particular relevance to patients with an acute STC. This has several advantages including the prediction of patient behavior. The approach also enables the clear definition of three self-care concepts, namely Self-Care First, Treatment Rate and Self-Care Behavior.

Between the two quantifiable concepts, it is evident that the Treatment Rate concept is the main self-care concept and thus of most significance to LMICs. This concept places emphasis on how individuals in LMICs can benefit from increased self-care practices through improved quality of life and welfare by reducing or avoiding the amount of lost wages that may result from not treating an STC. Moreover, this concept highlights individual gains in productive time that can be achieved through self-care, which eliminates the need to spend time seeking or engaging in primary healthcare services. Research demonstrates that time burden is strongly associated with socioeconomic status. Lower-income groups spend more time seeking care than higher-income groups due to increased travel, waiting and administrative times (47). Thus, self-medication may contribute to a reduction in healthcare disparities by providing individuals in LMICs with low-threshold access to healthcare without major general disruptions to daily routines.

The methodological framework for determining the value of self-care presented in this paper should be utilized with consideration of the following limitations. A key limitation of the systematic review is the fact that the majority of identified studies are in English language. However, no restrictions were placed on language and a thorough search of databases as well as websites of self-care associations was conducted in native languages. Additionally, more gray literature was identified than peer-reviewed literature. However, this was expected as many economic evaluation studies are not published and are often commissioned by government or non-prescription medicine associations.

The potential costs associated with the misuse of OTC and Rx medicines were not considered in this study. In fact, the role and contribution of pharmacists to the benefits and safety of self-medication is important and effective. Therefore, self-medication should be accompanied by pharmaceutical counseling whenever possible. If this is not possible in a given regional setting, a decision should be made on the basis of a risk-benefit assessment as to whether self-medication without pharmacist support or no treatment is the lower risk. Here, it was assumed that self-care is practiced by the patient under either the guidance of a pharmacist or by following product information. In general, non-existence of risks, either direct or indirect, when used correctly and/or if utilized without medical supervision, is among the criteria for the status of non-prescription medicine according to national or supranational regulatory authorities (5–9). These criteria limit the status of non-prescription treatment to self-diagnosable and self-limited conditions. The fact that a prudent further development of self-care within these defined limits could be clinically questionable is not supported by scientifically applicable findings and can therefore be disregarded at this point. This observation is particularly true in the setting considered here, where the alternative to self-treatment is often simply to do nothing at all.

Physician shortages and high costs for physician visits could be identified as the main hurdles for treatment in LMICs. Thus, a central prerequisite for self-care to improve health care in these regions is that people have access to clinically effective, safe and reliable OTC medicines along with investment in improving individuals' health literacy. Political will and an appropriate legal and economic framework in the countries can ensure this. The methodological framework presented in this paper gives political decision-makers, especially in low- and middle-income countries, a valid tool to assess the value and benefit of self-medication. However, the enablers that need to be addressed in order to further develop self-care vary greatly from country to country. The Self Care Readiness Index project recently conducted by the Global Self-Care Federation (GSCF) on the basis of surveys and analyses in 20 countries provides important insights with regard to the very different national and regional priorities (48). In addition to national and regional institutions, initiatives at WHO level are also crucial for improving the situation. On the basis of this knowledge, a framework for effective self-medication can be created.

## Data availability statement

The original contributions presented in the study are included in the article/supplementary material, further inquiries can be directed to the corresponding authors.

## Author contributions

All authors listed have made a substantial, direct, and intellectual contribution to the work and approved it for publication.
